# Quantitative relationships among high-throughput sequencing, cyanobacteria toxigenic genotype abundance and microcystin occurrence in bathing waters

**DOI:** 10.1016/j.scitotenv.2023.165934

**Published:** 2023-08-04

**Authors:** Magdalena Purker, Stefanie Dobrovolny, Michaela Kreuml, Peter Hufnagl, Alexander Indra, Rainer Kurmayer

**Affiliations:** a Austrian Agency for Health and Food Safety, Institute for Medical Microbiology and Hygiene - Center for Anthropogenic Infections, Department of Clinical Molecular Biology, Währinger Straße 25a, 1090 Vienna, Austria; b Austrian Agency for Health and Food Safety, Institute for Medical Microbiology and Hygiene - Center for Anthropogenic Infections, Department of Water and Hygiene, Währinger Straße 25a, 1090 Vienna, Austria; c Austrian Agency for Health and Food Safety, Institute for Food Safety, Department of Molecular Biology and Microbiology, Spargelfeldstraße 191, 1220 Vienna, Austria; d Austrian Agency for Health and Food Safety, Institute for Hydroanalytics, Wieningerstraße 8, 4020 Linz, Austria; e Austrian Agency for Health and Food Safety, Institute for Medical Microbiology and Hygiene, Währinger Straße 25a, 1090 Vienna, Austria; f Universität Innsbruck, Research Department for Limnology, Mondseestrasse 9, 5310 Mondsee, Austria and Universität Innsbruck, Innrain 52, 6020 Innsbruck

**Keywords:** EU bathing water directive, Cyanobacterial harmful algal blooms (CHABs), Cyanotoxins, HPLC-HRMS, Monitoring, Scum formation

## Abstract

Toxin-producing cyanobacteria pose significant threats to human and animal health if exposed during recreational activities in bathing waters. To better safeguard public health and reduce health risks during the bathing season, an effective monitoring and management strategy is required. Molecular tools used to monitor toxigenic cyanobacteria have been evaluated on the basis of the efficiency and applicability of the method used to (i) establish an early-warning monitoring strategy for EU bathing water sites using both targeted quantitative polymerase chain reaction (qPCR) and non-targeted high-throughput sequencing (HTS) genotype analysis and (ii) to compare the toxigenic potential of cyanobacteria with actual microcystin (MC) occurrence and concentrations. For this purpose, 16 bathing water sites were monitored according to the bathing water directive (BWD) of the European Union (EU) during the bathing season of the summer of 2020 in eastern Austria. The cyanobacterial community composition was analyzed through HTS and qPCR by targeting the microcystin synthetase B gene (*mcy*B), which indicates MC synthesis within the genera *Microcystis* and *Planktothrix*. Within the genus *Microcystis*, which was identified as the primary MC producer, the *mcy*B genotypes formed stable subpopulations that increased linearly in correlation with the total *Microcystis* population. Notably, the HTS cell equivalents assigned to *Microcystis* and *Planktothrix* correlated with the corresponding qPCR estimates of genotype abundance, which serves as a confirmation of the suitability of (semi)-quantitative sequencing through HTS. In addition to the elevated trophic state, reduced transparency, increasing water temperatures, as well as cyanobacterial HTS read numbers and *Microcystis* cell number equivalents per mL estimated through qPCR, were associated with positive MC samples. Therefore, in combination with the monitoring of standard environmental parameters, the use of HTS and qPCR techniques is considered highly useful to ensure the timely identification of health risks to recreational users, as mandated by the BWD.

## Graphical abstract

**Graphical Abstract:**
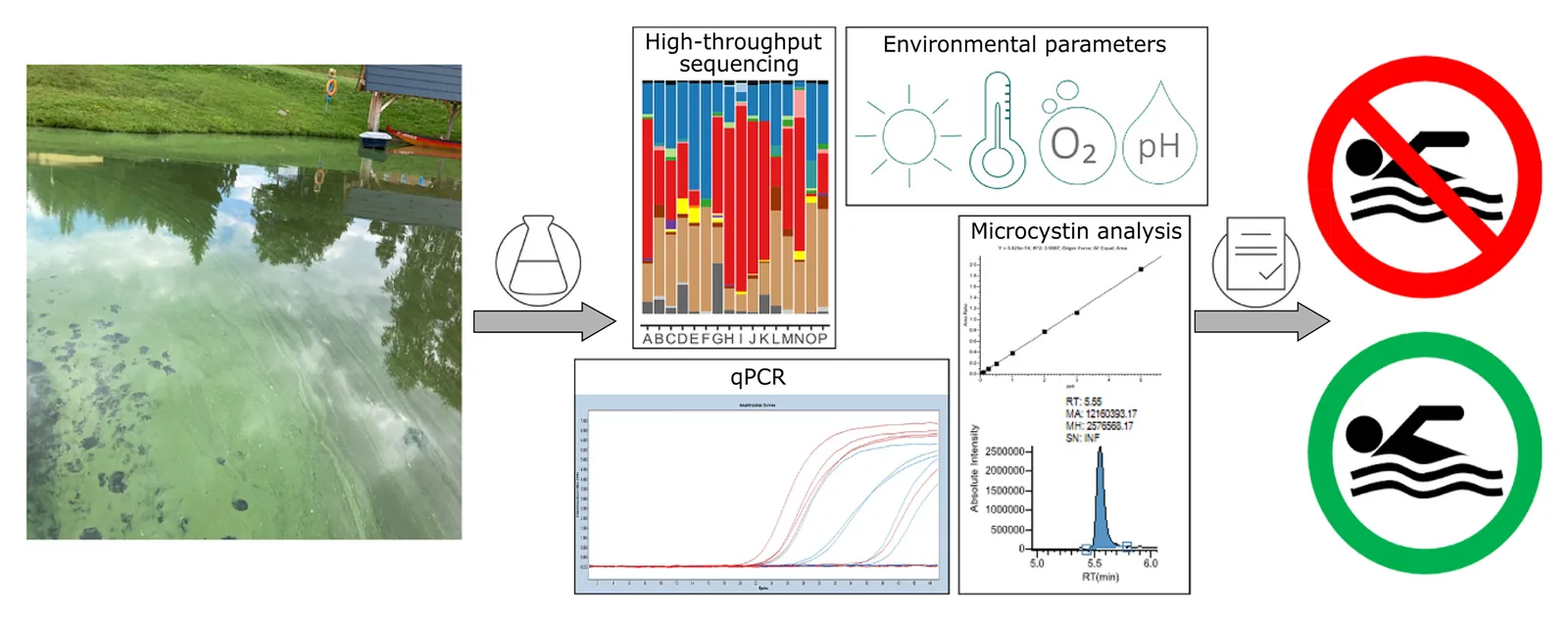

## 1 Introduction

Member states of the European Union (EU) are legally required to systematically monitor and assess bathing waters between June and August each year according to the Bathing Water Directive (BWD, 2006/ 7/EC). According to the BWD, bathing waters are defined as any kind of surface water which is used by people to bathe and not affected by a bathing prohibition. The BWD can assess bathing waters according to the number of colony-forming units (cfu) of intestinal enterococci and *Escherichia coli*, which are indicator species for fecal contamination. Additionally, any bathing waters where there may be a risk of exposure to cyanobacteria must be monitored and managed appropriately. Scums from cyanobacterial blooms can result from moderate cell numbers in the water column by accumulation on shorelines within a short time span, depending on various factors such as wind direction (Ibelings et al., 2021). In general, efficient and ad hoc monitoring will be of increasing importance in the near future. On the one hand, planktonic cyanobacteria are ecologically favored under climate warming conditions. On the other hand, it is predicted that, depending on global warming in the lowlands (e.g., the increasing frequency of heat waves during summer), the public needs for recreational activities in waterbodies will increase (Pröbstl-Haider et al., 2021).

Since the early 1980s, the toxigenic potential of cyanobacteria and their harmful effects on human and animal health have been studied (Chorus et al., 2000). Numerous publications have already reported cases of humans, pets (especially dogs), and livestock suffering from symptoms mentioned above after contact with waters containing cyanotoxins (e.g., Chorus et al., 2000, and references therein; Fastner et al., 2018; Mez et al., 1997). In addition, taste and odor compounds often produced by cyanobacteria can lead to the reduction of water quality and related ecosystem services such as the aesthetic value (Kaloudis et al., 2017). When humans are exposed to water contaminated with cyanobacterial toxins, they can suffer from various health problems such as skin irritations (e.g., rash and sores), gastrointestinal issues such as diarrhea, nausea, fever, vomiting, lethargy, paralysis, and even death (Centers for Disease Control and Prevention, 2021). High cyanotoxin concentrations can accumulate along the shoreline due to the wind-driven assembling of floating scums, endangering recreational users and pets during playing and splashing (Ibelings et al., 2014).

The detection and monitoring of cyanobacterial harmful algal blooms (CHABs) involve a combination of techniques and technologies (Srivastava et al., 2013). One widely used approach is through microscopic analysis (e.g., algal cell counting and calculation of the biovolume), which is used by the majority of EU member states (e.g., France, Germany, Hungary, Italy, and the Netherlands) to estimate the abundance of potential cyanotoxin producers (Chorus, 2012). Although it is time-consuming and relies on taxonomical expertise, this technique allows a direct insight into the cyanobacterial community composition (Ibelings et al., 2014). Not all cyanobacterial species can produce toxins, and because the toxic and non-toxic strains co-exist in the environment and are morphologically identical, they can only be distinguished based on their genotype (i.e., containing the genes resulting in cyanotoxin synthesis or not) (Capelli et al., 2017; Kurmayer et al., 2017). Fluorescence analysis is another commonly employed technique both in-situ and in the laboratory (e.g., used in the Netherlands) (Chorus, 2012) and uses the excitation/emission of fluorescent pigments such as chlorophylls and phycobilins (Padisak et al., 2021) to estimate the abundance of cyanobacterial cells in a water sample. Fluorescence analysis offers several advantages for cyanobacterial monitoring, including the simple handling and, in case of in-situ fluorescence probes, the timely detection of the presence of the cyanobacterial proportion within the phytoplankton community (Brient et al., 2008; van der Oost et al., 2010). This technique may help to gain a comprehensive understanding of the expected toxin concentration and supports rapid decision making for water managers. However, multiple factors, such as cell morphology (e.g., age, size, and volume), variability in the cellular pigment content, the presence of dissolved organic matter, including humic substances, and the heterogenic distribution of the algal cells (i.e., aggregation of single cells into colonies), may lead to an increase in the variability of the observed fluorescence signals (Bertone et al., 2018).

In contrast, molecular-based technologies for monitoring cyanobacterial blooms can be performed via high-throughput sequencing (HTS) and/or qPCR. The advantages of applying qPCR include (i) early warning, i.e., the early identification of toxigenic genotypes via a fast and sensitive approach, (ii) quantifying toxigenic genotypes via specific amplification, and (iii) understanding environmental factors leading to toxigenic genotype growth and seasonal variations in abundance (Pacheco et al., 2016; Padisak et al., 2021). Through HTS, a comprehensive list of potential toxin producers can be generated, whereas by using a targeted assay, such as qPCR, rare species may be overlooked (Casero et al., 2019). However, the detected genotypes identified through HTS are difficult to quantify (Shelton et al., 2022). Thus, a step forward could be to use HTS for monitoring the entire cyanobacteria community while quantifying specific toxigenic genotypes via qPCR.

Several studies have already applied HTS for monitoring CHABs (e.g., Casero et al., 2019; Eldridge et al., 2017; Fortin et al., 2015; MacKeigan et al., 2022; Parulekar et al., 2017; Tromas et al., 2017; Van Hassel et al., 2022). However, the combination of both HTS and qPCR methods together has been explored less frequently (but see Eldridge et al., 2017). Therefore, we implemented a monitoring approach which could benefit from both targeted and non-targeted genotype analyses. The objective of this study was to elaborate an early-warning monitoring system for bathing water sites to identify and quantify toxigenic cyanobacterial genotypes, with the aim to meet the requirements of the BWD. More specifically, we wanted to (i) identify the bathing waters which have the highest potential for cyanobacterial proliferation, (ii) determine the dominant microcystin (MC)-producing cyanobacterial genera, and (iii) pinpoint the environmental parameters related to CHAB occurrence. Thus, we applied a combined approach including HTS to characterize the cyanobacterial community composition, quantify the toxigenic genotypes based on qPCR, analyze the MC concentrations, and document the related environmental variables.

## 2 Materials and methods

### 2.1 Sampling and bathing site description

According to Annex III of BWD, bathing water profiles must i) address the physical, geographical, and hydrological characteristics of a bathing water, ii) identify and assess sources of pollution, and iii) evaluate the potential of phytoplankton proliferation. The bathing water profiles must be reviewed and adapted to possible natural changes in the ecosystem at least every 4 years. Although no standardized methods are proposed in the BWD, the methodology used to assess the potential for the proliferation of cyanobacteria is important. An overview of the site description, the methodologies used to classify the cyanobacterial bloom risk, and the final risk classification for the 16 study sites are given in Table S1. Whilst some of the bathing water profiles have been established based on comprehensive monitoring of the dominant cyanobacterial genera, as well as the trophic condition and cyanotoxin concentration (e.g., I, J), others have been assigned based solely on visual inspections (e.g., A, B, E, F, H, O). In summary, the risk assessment of the bathing water profiles indicates that five of the sampling sites present a high risk for developing cyanobacterial blooms (A, G, H, I, J), three sites indicate no risk (B, E, F), and eight bathing water sites (C, D, K, L, M, N, O, P) are considered to have a medium risk level.

During the bathing season of 2020, 80 planktonic samples were taken from shore from 16 different EU bathing water sites (abbreviated with the synonyms A–P) located in eastern Austria (Table S1). Five samples were taken per bathing site every 2 or 3 weeks during the study period (1st sampling date in early June: 2/3 June 2020, 2nd sampling date in mid-June: 15/16/17 June 2020, 3rd sampling date in early July: 6/7/8 July 2020, 4th sampling date at the end of July: 27/28/29 July 2020, and 5th sampling date during mid- August: 17/18/20 August 2020) according to the BWD protocol. Water sampling was carried out at those sites where the number of recreational users was highest. Therefore, sampling points were located on jetties and beaches with the highest bathing activity.

All samples were collected from the littoral zone, at a depth of 30–50 cm, using a crucible tong. Overall, a total of 950 mL of water was sampled, including 250 mL for the MC analysis, 600 mL for DNA extraction, and 100 mL for total phosphorus (TP) determination. For fixation of the TP sample, 1 mL of HNO_3_ was added immediately. All water samples were transported to the laboratory within 24 h, ensuring they were stored in a cool and dark environment throughout the journey.

### 2.2 Water physics, chemistry and chlorophyll-a in-situ fluorescence

Dissolved oxygen (O_2_), pH, and water temperature (T) were measured using an FDO® 925-P and SensoLyT® 900-P probe (WTW, Weilheim, Germany). Secchi depth was determined with a Secchi disc with an accuracy of 10 cm. The TP concentration was determined by inductively coupled plasma-mass spectrometry (ICP-MS) according to ÖNORM EN ISO 17294-1:2007 and ÖNORM EN ISO 17294-2:2007 at the AGES Institute for Hydroanalytics (Linz, Austria). For the analysis of total chlorophyll (TChl) and cyanobacteria-specific chlorophyll (CChl), an in-situ spectrofluorometer was used (AlgaeTorch, bbe moldaenke, Schwentinental, Germany). Each measurement was repeated three times, and the average value was used for analysis. Whilst TChl indicated the biomass of the total algal community from the fluorescence of chlorophyll-a, CChl indicated the proportion of cyanobacteria from the fluorescence of phycocyanin.

### 2.3 Microcystin analysis

For the determination of MC structural variants (MC-LR, MC-RR, MC-YR and MC-AspRR), a high-resolution mass spectrometer (HRMS), i.e., a QExactive Orbitrap Mass Spectrometer (Thermo Fisher Scientific, Waltham, Massachusetts, USA), was used, coupled with chromatographic separation by high-performance liquid chromatography (HPLC-HRMS). Extraction and MC analysis were performed at the AGES Institute for Hydroanalytics (Linz, Austria). For the analysis of the dissolved MCs, 1.5 mL of the shaken and filtered (0.45-μm cellulose-acetate filter) water sample was used. Total MCs (including both intracellular and dissolved fractions) were extracted using cell lysis via repeated freezing and thawing of the samples (ISO 22104:2021). For injection, 1.5 mL of the cell-lysed sample was filtered and spiked with the internal standard. Routinely, the non-naturally occurring molecule atrazine-d5 was added as internal standard to the sample to control for the injection volume. For the standard curves, the concentration of the respective MC variant ranged from 0.05 to 5.00 μg L^−1^, which was also compared with theinternal standard. For chromatographic separation, a Waters XSelect HSS T3 (150 × 2.1 mm, pore size 3.5 μm) column with a gradient elution of solvent A (HPLC H_2_O) and solvent B (C_2_H_3_N with 0.1 % CH_2_O_2_), for a duration of 14.1 min was used. The injection volume was 100 μL. The retention times for the analyzed MC variants are summarized in Table S2. The software Xcalibur™ (version 4.3, Thermo Fisher Scienti-fic, Waltham, Massachusetts, USA) was employed to commission the mass spectrometer and the HPLC. Finally, the data were evaluated with TraceFinder™ (version 3.2, Thermo Fisher Scientific, Waltham, Massa-chusetts, USA). The confirmation of individual MCs was made through LC-HRMS. The quantification was done from the exact mass trace (HRAM–high-resolution accurate mass), extracted from the HR-fullscandata and the triggered MS2-spectra considering the internal standards. The calculated values of the limit of detection (LOD) and limit of quantification (LOQ) of the respective MC variants are summarized in Table S2.

### 2.4 DNA extraction and quantification

Depending on the amount of particulate matter in the water sample, a volume between 50 and 600 mL was filtered manually through a Sterivex™-GP 0.22-μm filter (Millipore, Billerica, Massachusetts, USA) and frozen at −20 °C until DNA extraction. Genomic DNA was extracted using the DNeasy® PowerWater® Sterivex™ Kit (Qiagen, Hilden, Germany) following the manufacturer’s protocol. After elution of the DNA from the spin column by adding two times 50 μL of DNA-free PCR-grade water, the DNA concentration was determined by a Qubit 2.0 Fluorometer (Thermo Fisher Scientific, Waltham, Massachusetts, USA) using the dsDNA BR Assay Kit and stored at −20 °C.

### 2.5 qPCR for Microcystis and Planktothrix microcystin genotype quantification

For the evaluation of the MC-producing potential within the bloom-forming cyanobacterial genera, *Microcystis* and *Planktothrix*, Taq Nuclease assays (TNA) were performed, targeting the subpopulation of *mcy*B genotypes indicative of MC synthesis, as well as the phycocyanin intergenic spacer region (PC-IGS) quantifying the total population (Table 1), (Kurmayer and Kutzenberger, 2003; Kurmayer et al., 2011). The reaction mix and the PCR conditions for qPCR are described in Kurmayer and Kutzenberger (2003) and Ostermaier and Kurmayer (2010), using a LightCycler® 480 Instrument (Roche, Basel, Switzerland). The *Microcystis* qPCR probes were equipped with BBQ as quencher, whereas for the *Planktothrix* qPCR probes, Tamra was used as fluorescence dye. The PCR products were further analyzed by electrophoresis in 2 % agarose gel.

To calibrate the qPCR results, clonal cultures of *Microcystis* (*M. aeruginosa* Hofbauer) and *Planktothrix* (NIVA-CYA 126/8) were grown in BG11 medium at 20 °C with 50 μE m^−2^ s^−1^ irradiation (12 h:12 h) under maximum growth rate conditions following the turbidostatic principle. Cells were harvested during the logarithmic growth phase, and aliquots were enumerated for cell numbers (*Microcystis*) or biovolume (*Planktothrix*) under the microscope (as described previously, SOP 7.2, in Kurmayer et al. (2017); for details, see Suppl. Information). In parallel, DNA was extracted from harvested cells using Sterivex filters exactly as for the environmental samples (see Chapter 2.4). From the DNA extracts, 10-fold dilution series (until a dilution of 10^−7^) were prepared, and qPCR was performed in triplicate. For each strain, a linear calibration curve (Fig. S1) was generated through relating the obtained mean Ct-value to the logarithm of the corresponding cell equivalents or biovolume content (Kurmayer et al., 2017, SOP 7.2). Furthermore, the linear regression equation, the coefficient of determination (r^2^), and the amplification efficiency (AE) were determined for each qPCR assay (Table S3). The theoretical levels of detection (LOD) and quantification (LOQ) (Table S4) were defined as given in Zupančič et al. (2021).

### 2.6 PCR for HTS

For HTS, environmental DNA extracts (with a DNA concentration between 1 and 13.8 ng μL^−1^) were, if possible, diluted to a concentration of 5 ng μL^−1^. Overall, two PCR assays were performed, one for the 16S rDNA V3–V4 region of bacteria in general and one for the 16S rDNA V3–V4 region of cyanobacteria only. For amplifying bacteria, the primer pair Bakt_341F/Bakt_805R (Herlemann et al., 2011), and for cyanobacteria, the primer pair 515F/CYA781R(a) and CYA781R(b) (Nübel et al., 1997; Turner et al., 1999) were used (Table 1 and Suppl. Information). Library preparation and paired-end sequencing (2 × 250 bp) for amplified 16S rDNA were carried out on a MiSeq platform (Illumina, Sand Diego, CA, USA) according to the manufacturer’s protocol, with minor modifications (i.e., 30 instead of 25 cycles, no heating step, and no incubation step for 5 min in ice water) and under standard conditions at the Institute for Food Safety (AGES) (Dobrovolny et al., 2018).

### 2.7 Sequence analysis and data processing

The raw sequence fastq files were processed in R (version 4.2.2; R Core Team, 2022) using the package Divisive Amplicon Denoising Algorithm 2 (DADA2), which was introduced by Callahan et al. (2016) (version 1.20.0). The raw sequences generated for this study were submitted to the NCBI Raw Sequence Read Archive (SRA) under accession number PRJNA913666. During quality trimming, truncation lengths were set to 250 and 220 bp for the bacterial forward and reverse reads, respectively. For the cyanobacterial forward and reverse reads, the truncation lengths were set to 250 and 210 bp, respectively. The quality score was set to 2 (implying the trimming of the sequence at the first nucleotide with a quality of 2 = statistically one error in 100 bp).

By using the bacterial primers 11,293,104 sequences were obtained, of which 3,815,730 passed the bioinformatic quality filtering. With the cyanobacterial primers, 11,680,571 sequences were obtained, of which 9,397,107 remained. Sequences were further clustered into Amplicon Sequence Variants (ASVs) (= no dissimilarity threshold) and taxonomically assigned to the SILVA SSU reference database (version 138; Quast et al., 2013). All ASVs assigned to chloroplasts, mitochondria, or archaea were excluded from further analysis, resulting in 12,861 ASVs (bacterial) and 1876 ASVs (cyanobacterial), respectively.

To facilitate the comparison of sequencing results across samples, read numbers had to be rarefied, resulting in the same number of reads for each sample (Cameron et al., 2020). By using the iNEXT package (version 2.0.20; Hsieh et al., 2016), rarefaction curves for each sampling date and primer system were created to estimate the loss of information by choosing the appropriate rarefying threshold (9484 bacterial reads, 2519 cyanobacterial reads) (Figs. S2 and S3). Rarefaction was performed using vegan’s *rrarefy* command (version 2.5–7; Oksanen et al., 2022). Furthermore, the R-package vegan was also used for calculating the alpha diversity indices (Fig. S4). The ASVs belonging to the phylum *Cyanobacteria* were additionally blasted against a customized database (containing 51 cyanobacterial genotypes), using the R-package rBLAST (version 0.99.2; Hahsler and Nagar, 2019). For visualization, the R-package ggplot2 (version 3.3.5; Wickham, 2016) was employed.

### 2.8 Multivariate statistical analysis of environmental variables

Principal component analysis (PCA) was used to explore the relationship between MC occurrence and other variables, such as water temperature, O_2_, pH, Secchi depth, TP concentration, fluorescence measurements, as well as *Microcystis* cell equivalents based on qPCR. Furthermore, PCA was applied to analyze MC-positive samples according to their MC-variant composition. First, to prevent autocorrelation, environmental variables that showed strong correlations with each other were excluded from the analysis (e.g., TChl vs. CChl and qPCR vs. HTS). Second, seasonal adjustment was obtained by subtracting the mean value per variable for each sampling date from the corresponding value in the original dataset, as outlined by Hörmann and Unkel (2015).

For visualization of the PCA biplot, the R-package factoextra (version 1.0.7, Kassambara and Mundt, 2020) was used, whereas significance was tested with the R-package PCAtest (version 0.0.1, Camargo, 2022) by calculating 100 bootstrap replicates. The analysis revealed a statistical significance for the PCA models (*p* < 0.01).

## 3 Results

### 3.1 Cyanobacterial community composition

During the study period, the phylum Cyanobacteria increased in abundance relative to other bacterial phyla (Fig. S5).The observed increase in the dominance of the phylum Cyanobacteria relative to other heterotrophic bacterial phyla throughout the bathing season aligns with the expected growth behavior and reinforces the validity of HTS as a semi-quantitative approach (Fig. S5). Using HTS with bacterial primers, the phylum Cyanobacteria accounted for 149,604 reads, which corresponded to approximately 20 % of all bacterial reads. More details regarding the overall bacterial community composition can be found in Fig. S5. Using universal bacterial primers resulted in the identification of 587 cyanobacterial ASVs, whereas the use of cyanobacterial primers yielded a total of 624 ASVs assigned to the phylum Cyanobacteria. The most abundant cyanobacterial ASV during this study was assigned to *Microcystis*, i.e., ASV5 with bacterial primers (12,842 sequences) and ASV2 with cyanobacterial primers (15,110 sequences), respectively. However, as indicated in Table 2, *Cyanobium* emerged as the predominant cyanobacterium throughout the sampling season, comprising >50 % of the assigned cyanobacterial reads. In general, other potential MC-producing Cyanobacteria such as *Planktothrix, Dolichospermum, Aphanizomenon*, and *Nostoc* exhibited lower levels of abundance.

The supplementary BLASTn search conducted for the obtained ASVs generally validated the taxonomic assignments made using the SILVA database. Nonetheless, in the case of six (bacterial) and seven (cyanobacterial) ASVs, the BLASTn algorithms identified *Woronichinia naegeliana* 0LE35S01 (accession number AJ781043.1) as the genus, contrasting with the SILVA-assigned genus *Snowella*. Consequently, 7646 bacterial and 7797 cyanobacterial reads, respectively, were reassigned to *Woronichinia* rather than *Snowella*. Furthermore, cyanobacterial ASV1297 (including 5334 reads) automatically assigned to *Aphanizomenon MDT14a* were reassigned to *Dolichospermum planctonicum* 1tu28s8 (accession number AJ630430.1).

### 3.2 Quantification of the relationship between HTS read numbers and microcystin genotype abundance

**Fig. 1:**
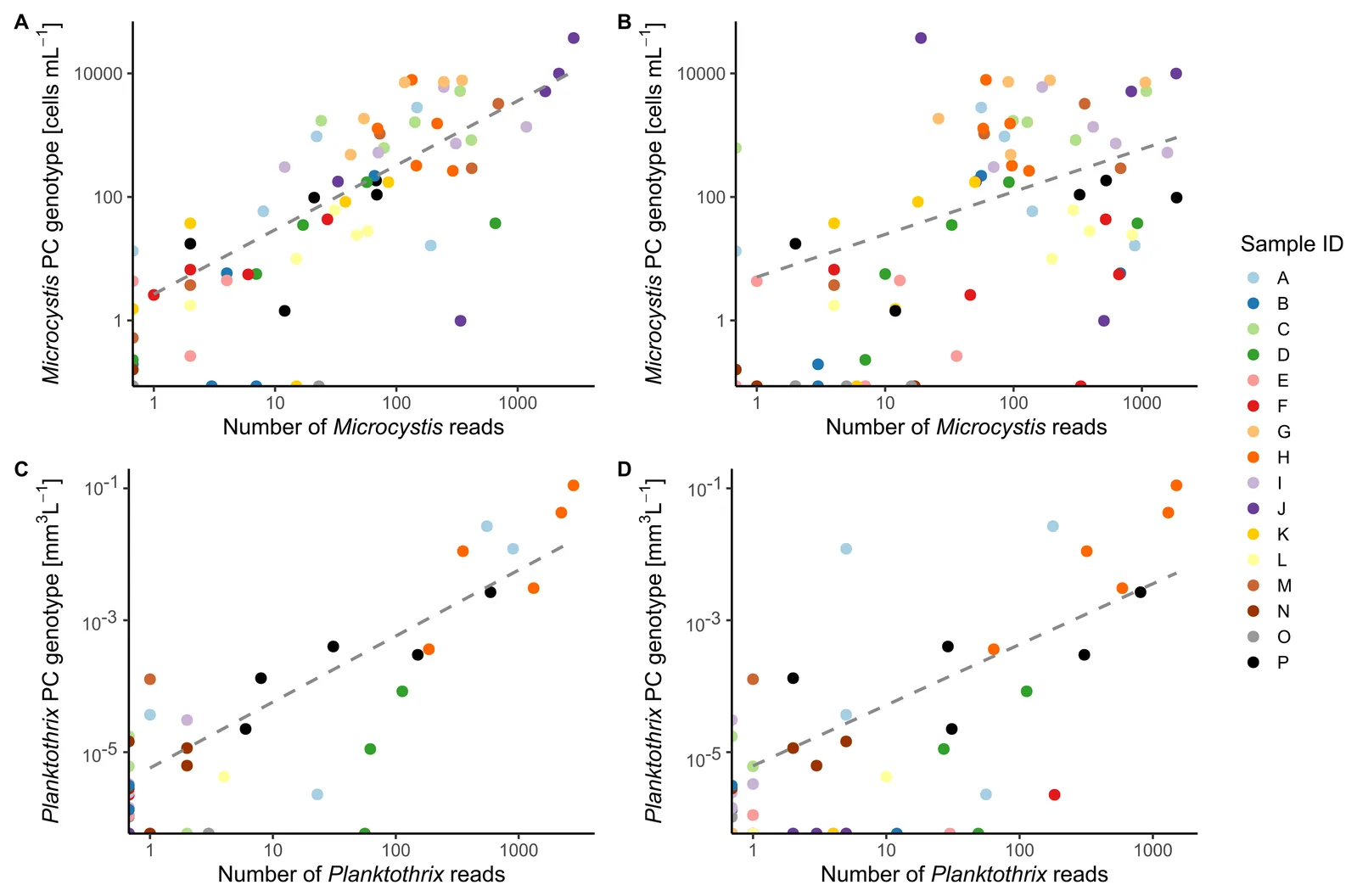
Correlations between qPCR-derived cell numbers or biovolume (y-axis) and rarefied HTS read numbers (x-axis) for the two abundant bloom-forming cyanobacterial genera *Microcystis* and *Planktothrix*. (A, B): Cell number equivalents per mL recorded by qPCR (PC – *Microcystis*) plotted against *HTS* read numbers assigned to *Microcystis* using (A) the bacterial primers (y = 0.05 + 1.2×) and (B) the cyanobacterial primers (y = 0.27 + 0.83×). (C, D) Biovolume in mm^3^ L^−1^ recorded by qPCR (PC – *Planktothrix*) plotted against HTS read numbers assigned to *Planktothrix* using (C) the bacterial primers (y = -6 × 10^−4^ + 2.8 × 10^−5^x) and (D) the cyanobacterial primers (y = -0.00059 + 4.5 × 10^−5^x); *N* = 80.

To investigate the relationship between the abundance of potential MC-producing cyanobacteria and the number of HTS reads, a separate quantification of *Planktothrix* and *Microcystis* was performed using qPCR alongside HTS. Overall, the rarefied HTS read numbers inferred from bacterial primers (Fig. 1A and C) showed a high linear correlation with the qPCR results (Spearman’s rho =0.81 and 0.71, *p* < 0.001 for *Microcystis* and *Planktothrix*, respectively). The HTS read numbers inferred from cyanobacterial primers (Fig. 1B and D, Spearman’s rho =0.56 and 0.58) revealed a lower, albeit significant correlation (*p* < 0.001). Thus, our findings show that the numbers of *Microcystis* and *Planktothrix* HTS read numbers correlated with cell numbers and biovolume estimated by qPCR, respectively.

As the highest correlation observed between HTS reads inferred from bacterial primers and the abundance of toxigenic cyanobacteria, as well as the comparable cyanobacterial genus composition obtained from using both HTS primer pairs (Table 2), further analysis focused solely on the results obtained using the bacterial primers.

**Fig. 2:**
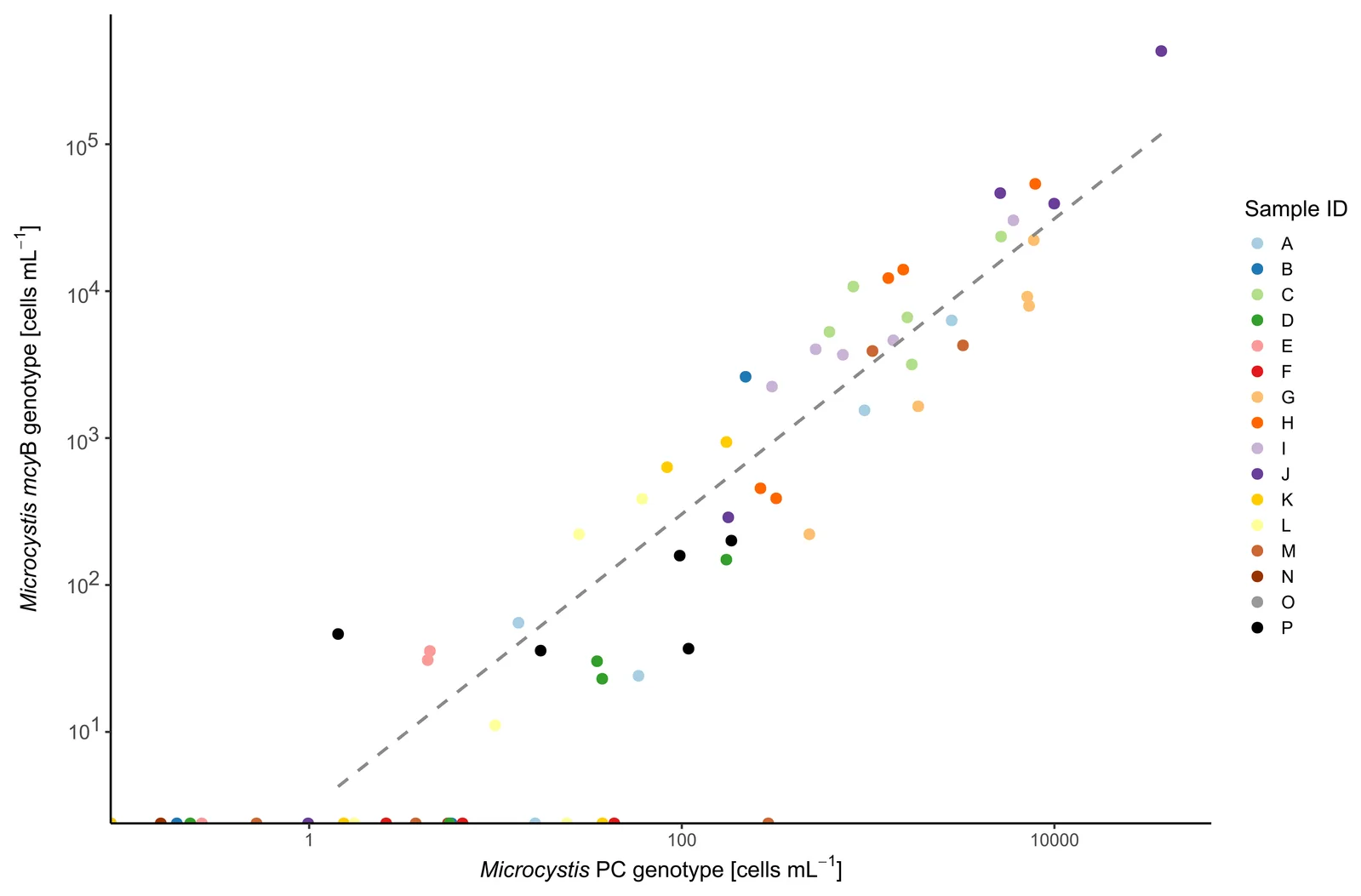
Relationship between *Microcystis* PC and *mcy*B genotypes (in cell number equivalents per volume). N = 80; y = 0.018 + 1.1×.

For most samples (*N* = 46, 58 %), both PC and *mcy*B genotypes of *Microcystis* were amplified by qPCR. Notably, a significant linear correlation between *Microcystis* PC and *mcy*B genotype cell numbers was observed (Spearman’s rho = 0.91), with *mcy*B genotype cell number equivalents per mL ranging from 11 to 2614 (Fig. 2). For 19 samples (23%), only the PC genotype (cell number equivalents per mL ranging from 0.16 to 291) was observed whereas no signal was obtained via the *mcy*B qPCR assay. For 15 samples (19 %), neither a signal for PC nor *mcy*B in *Microcystis* was obtained.

For *Planktothrix*, in 28 samples (35 %), a PC signal was detected via qPCR, with calculated biovolumes ranging from 1.04× 10^−6^ to 0.11 mm^3^ L^−1^. In contrast to PC, a low number of *mcy*B-positive samples (*N* = 10, 13 %) was observed, which originated from three sampling sites (H, N, P). The calculated *mcy*B biovolumes ranged from 2.38 ×10^−5^ to 1.95 × 10^−3^ mm^3^ L^−1^. As for *Microcystis*, a positive relationship between PC and *mcy*B genotype in *Planktothrix* was observed using non-linear rank order correlation (Spearman’s rho = 0.59, p < 0.001, Fig. S6).

### 3.3 Microcystin concentrations in EU bathing waters

In general, MC was detected throughout the study period. At least 1 MC structural variant was detected in 19 samples, accounting for one third of the 64 samples collected. Among the 8 MC-positive sites (A, B, C, G, H, I, J, K), the most commonly detected MC variant was MC-RR (in 84 % of positive samples), followed by MC-LR (79 %) and MC-YR (31 %).

**Fig. 3:**
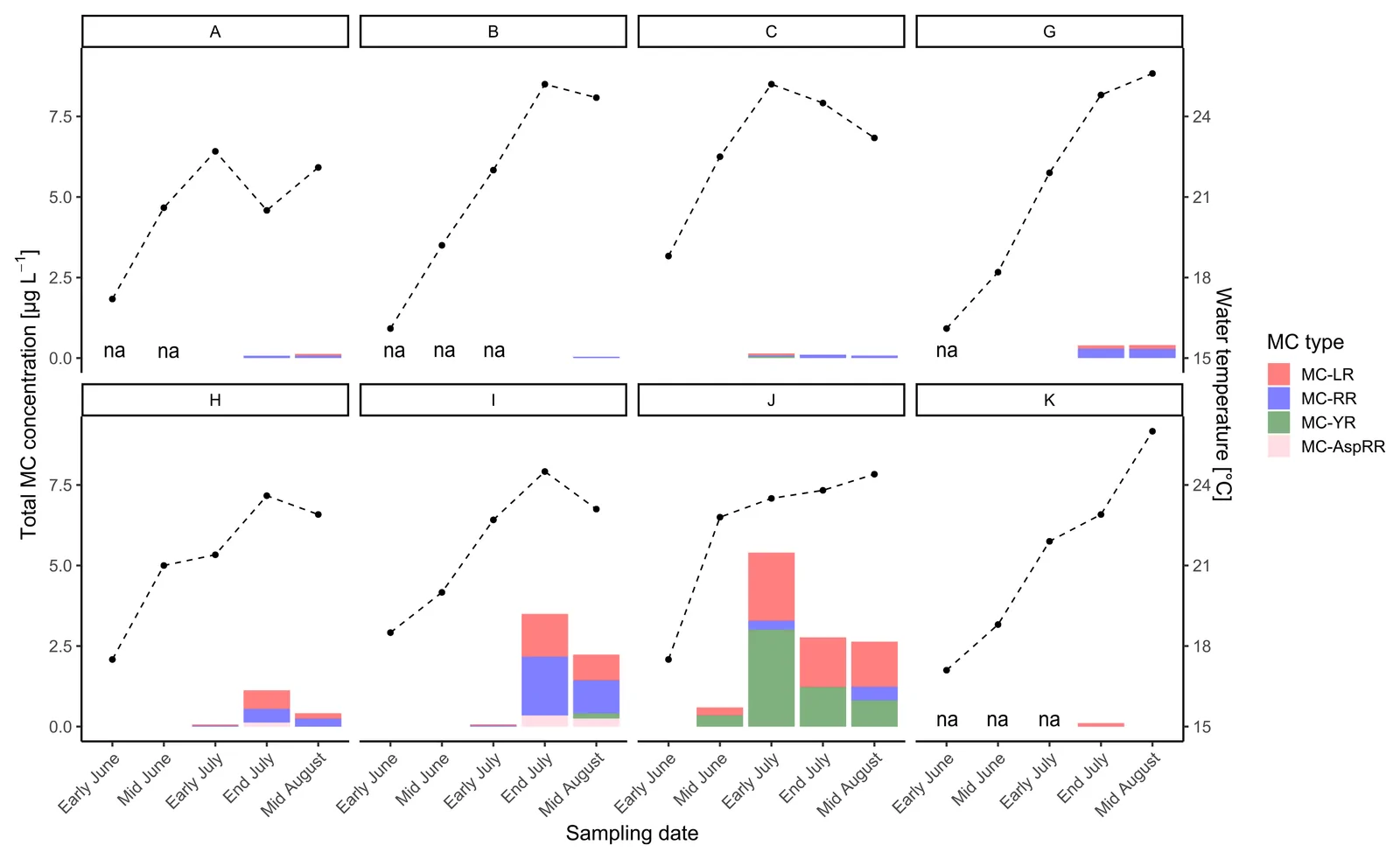
Microcystin concentrations recorded from 16 EU-bathing water sites (indicated by sample ID) located in the east of Austria during the bathing season in the summer of 2020. The stacked bars represent the concentration of the individual MC structural variants (left y-axis). The corresponding water temperature is indicated by the black dashed line (right y-axis). No MC data are available for samples marked by “na”.

The MC variant MC-AspRR could only be detected in 3 out of 19 positive samples (16 %). Fig. 3 demonstrates the variation among MC variants throughout the sampling season and sampling sites. Specifically, sampling site I showed the highest MC-RR concentrations toward the end of July and continuing until mid-August, with record values of 1.82 and 1.02 μg L^−1^, respectively, corresponding to the maximum (max.) measured MC-RR concentration. Sampling site J exhibited high concentrations of MC-LR and MC-YR. In detail, the max. Concentration of MC-LR, measuring 2.11 μg L^−1^, was observed in early July, followed by a slight decrease as the season progressed to 1.54 and 1.40 μg L^−1^. Regarding MC-YR, the max. Concentration was detected during early July (3.01 μg L^−1^). Aside from sampling site J, MC-YR was only detected at minor concentrations in the sampling sites C and I (< 0.5 μg L^−1^).

Bathing site J had the longest period of MC production, as evidenced by the recording of a total MC concentration of 0.6 μg L^−1^ already in mid-June (Fig. 3). In early July (third sampling date), the max. MC concentration during the study period was observed (5.4 μg L^−1^), followed by 2.8 μg L^−1^ at the end of July (fourth sampling date) and 2.6 μg L^−1^ for the last sampling date of mid-August. For all other sites, MC was not detected before the third sampling date. During the period of early July until mid-August, MC was detected at sites C (0.07–0.14 μg L^−1^), H (0.07–1.13 μg L^−1^) and I (0.08–3.5 μg L^−1^). During late-July and mid-August MC was detected at sites A (0.07, 0.13 μg L^−1^) and G (0.39, 0.4 μg L^−1^). At sites B and K, MC was detected only once (in mid-August and late-July, respectively), with 0.04 and 0.11 μg L^−1^, respectively.

### 3.4 Relationship between potential microcystin production and microcystin concentrations in EU bathing waters

**Fig. 4:**
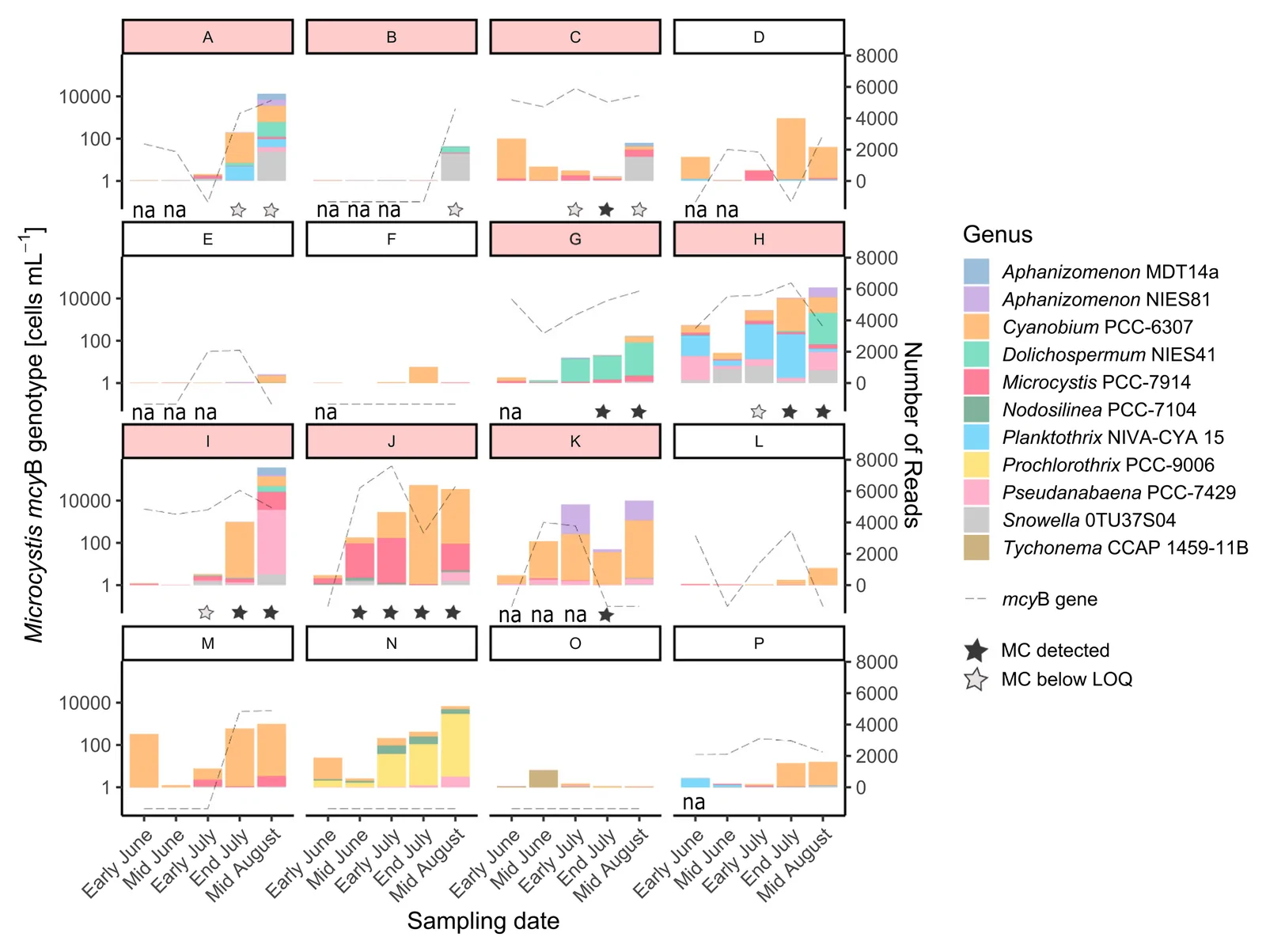
Relationships among abundance of cyanobacterial genera, *Microcystis mcy*B genotype abundance (qPCR), and microcystin occurrence at 16 EU bathing water sites (as indicated by sample ID) during the bathing season in the summer of 2020. The barplots represent the cyanobacterial genera (identified by HTS). The black dashed line represents the *mcy*B genotype cell equivalents of *Microcystis*. For clarity, the *mcy*B genotypes indicative of MC production in *Planktothrix* are not shown (i.e., observed only once at sample site H at the end of July with 0.0008 mm^3^ L^−1^ biovolume equivalents). MC occurrence (MC > LOQ) is indicated by a black star, and the samples with MC > LOD but < LOQ are marked with a grey star. No MC data are available for samples marked by “na”. The red boxes highlight MC occurrence.

In general, a significant positive correlation was observed between the calculated *mcy*B genotype cell numbers of *Microcystis* and the total MC concentration (Spearman’s rho = 0.68, *p* < 0.001) (Fig. S7). Samples that tested positive for MC typically contained the *mcy*B genotype, which is specific for *Microcystis*, with cell equivalents ranging from 288 to 430,887 per mL). The max. Cell numbers of the *mcy*B genotype were recorded at site J during early July (Fig. 4). The only exception was found for sample K, where 0.11 μg L^−1^ MC-LR was detected, but the corresponding *mcy*B qPCR was negative. The influence of *Planktothrix* on MC production was considered to be of minor significance as only two MC-positive samples (Sample H from the third and fourth sampling dates) tested positive for the *mcy*B of *Planktothrix*. In these samples, the biovolume equivalents for *Planktothrix* were 0.0002 and 0.0008 mm^3^ L^−1^, respectively.

For a few sites, *mcy*B was not detected throughout the sampling season (F, N, O), whereas other sampling sites (C, G, H, I) showed consistently high numbers of the *Microcystis mcy*B genotype. Among the other sites, a variable abundance of the *mcy*B genotype was observed. At site J, a pronounced increase in *mcy*B cell number equivalents (from 0 to 39,422 cell equivalents per mL) was observed from early to mid-June, while from early July to the end of July, the *mcy*B cell numbers declined from 430,887 to 289 cell equivalents per mL. In most cases, a distinct increase in *mcy*B cell number equivalents per mL of *Microcystis* was observed toward the end of the sampling season (e.g., sites A, B, G,M). Calculating the average of all sites, the mean *mcy*B genotype cell equivalents per mL of *Microcystis* increased from 2465 (standard deviation (sd) = ± 7341 cell equivalents per mL) during the first two sampling dates (early until mid-June) to 6300 (sd = ± 13,326 cell equivalents per mL) during the last two sampling dates (end July and mid-August).

### 3.5 Relationships of physico-chemical characteristics and chlorophylla in-situ fluorescence with microcystin concentrations

On average, T was low during the first sampling period (17.2 °C) and high during mid-August (23.7 °C). Max. T occurred during the end of July at site P and during mid-August at site K (max – mean (±sd) - min = 26.0–21.3 (± 3.2) - 10.6 °C). The min. T was measured during the second sampling date at bathing site E. No significant correlation between T and MC concentration was observed (r^2^ = 0.04, *p* > 0.05), although MC was often detected above a T of 22.8 °C (Fig. 3).

The max. TP concentration was recorded at site C during the third sampling date (89.5–30.6 (± 23.9) - 0 μg L^−1^). The MC-positive samples were found between a TP concentration of 11.2 and 81.8 μg L^−1^. No correlation between TP and MC concentration was observed (r^2^ = 0.01, *p* > 0.1). Nevertheless, the TP concentration correlated with TChl (r^2^ = 0.48, *p* < 0.001, Fig. S8A) and CChl (r^2^ = 0.19, p < 0.001, Fig. S8B).During this study, the max. TChl was measured at site C during the third sampling date (128.3–29.6 (± 30.7) - 0.1 μg Chl-a L^−1^), whereas the max. CChl was measured at sampling site J during mid-August (70.7–8.6 (± 13.2) - 0 μg Chl-a L^−1^). The minimum TChl and CChl fluorescence concentrations recorded from MC-positive samples were 4.7 and 0.6 μg Chl-a L^−1^, respectively (i.e. at site I during end-July and site B during mid-August, respectively). Vice versa, the max. Concentrations for MC-positive samples recorded were 128.3 and 70.7 μg Chl-a L^−1^, respectively, at site C during end July and J during mid-August (equal to the max. TChl and CChl values observed during this study). A significant correlation was found between the number of cyanobacterial HTS reads and TChl as well as between cyanobacterial HTS reads and CChl (r^2^ = 0.19, p < 0.001, Fig. S8C and r^2^= 0.44, p < 0.001, Fig. S8D). Furthermore, the total MC concentration was significantly correlated to CChl (Spearman’s rho = 0.59, p < 0.001, Fig. S9). No correlation between MC concentration and TChl was observed (*p* > 0.001).

**Fig. 5:**
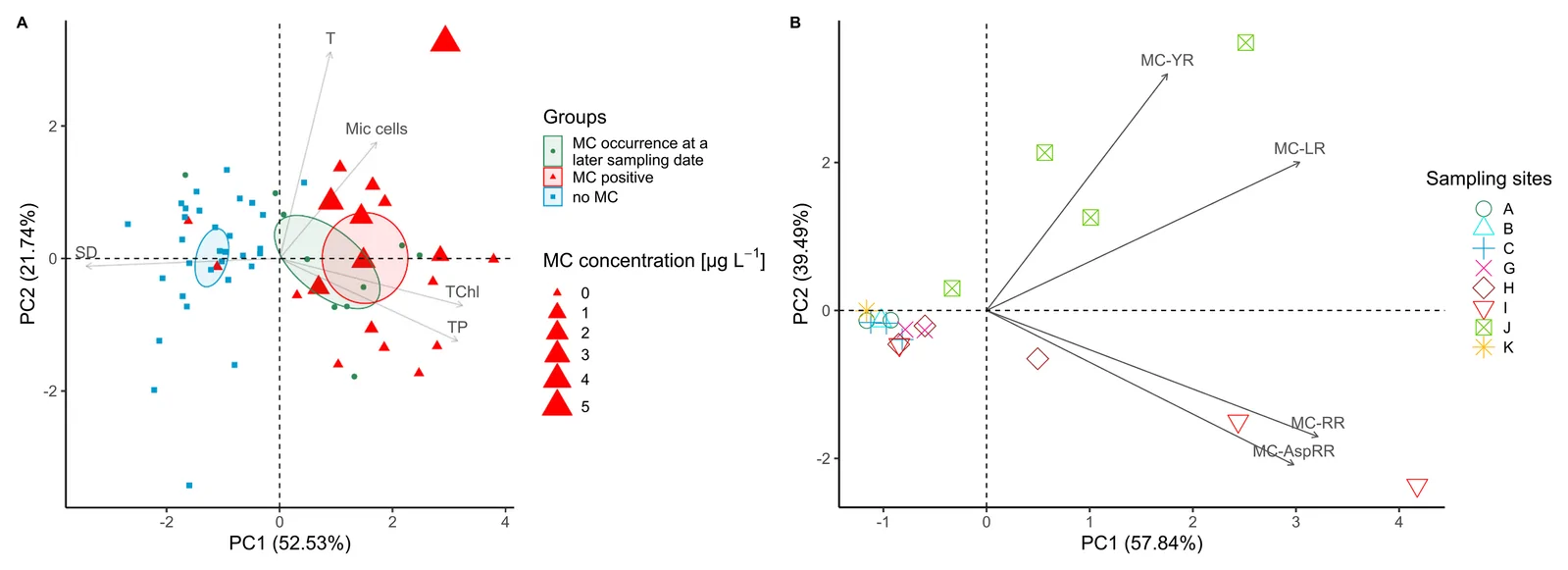
Illustration of the ordination of samples by principal component analysis (PCA). (A): PCA relating MC concentrations to environmental conditions including sum parameters and *Microcystis* PC genotype cell numbers: T = water temperature, SD = Secchi depth, TP = total phosphorus, TChl = total chlorophyll, Mic cells = cell equivalents of *Microcystis* PC genotype. The size of the symbols of each sample is relative to the recorded MC concentration. The confidence ellipses are plotted around the group means. (B): PCA of MC-positive samples with focus on MC variant composition.

To investigate the environmental characteristics associated with the occurrence of total MC, a PCA was performed (Fig. 5A). The variables TChl (variable loading = 0.54), TP (0.53) and SD (-0.57) had a significant influence on the first ordination axis PC1 (*p* < 0.01), explaining 52.5 % of the total variation. The second axis PC2 could only explain 21.7 % of the total variation (*p* > 0.01) and was mostly influenced by T (0.81) and PC genotype cell equivalents of *Microcystis* (0.46). Samples without MC (colored in blue) clustered in the left part of the ordination diagram. With only two exceptions, the samples of MC-positive sampling sites (represented by green or red symbols) were typically positioned in the right half of the biplot. Specifically, the two samples that tested positive for MC (from sampling site K on the fourth sampling date:0.11 μg L^−1^; from sampling site B on the fifth sampling date: 0.036 μg L^−1^) clustered together with all other samples of MC-negative sites. In general, the ordination of samples from MC-positive sites exhibited a positive correlation with reduced SD, increased concentrations of TP, TChl, and CChl, as well as with higher cell number equivalents of the *Microcystis* PC genotype. Additionally, a second PCA was calculated, focusing only on MC-positive samples and the relationship of MC variants (Fig. 5B). Both PC axis 1 and 2 were statistically significant (p < 0.01), whereas PC1 explained 57.8 % of the variation and was significantly loaded by MC-RR (0.57), MC-LR (0.54), and MC-AspRR (0.53). Contrarily, PCA2 explained 39.5 % of the variation and was primarily influenced by MC-YR (-0.69). The observed clustering identified a high dependency of MC variant occurrence on sampling sites. For example, samples from site J primarily clustered in the upper right half of the biplot, whereas samples from site I primarily grouped within the lower left half.

## 4 Discussion

### 4.1 Insights gained from the combination of HTS, qPCR and MC analyses

The objective of this study was to develop an early-warning monitoring system for bathing water sites, enabling the identification and quantification of toxigenic cyanobacterial genotypes through a combination of targeted (qPCR) and non-targeted (HTS) genotype analyses. Whilst qPCR-based targeted analysis offered quantitative estimates of specific genotypes indicative of MC synthesis, non-targeted analysis was the only method capable of providing insights into the general abundance of toxigenic cyanobacteria. The term “toxigenicity” has been defined as either the presence of genes indicative of toxin synthesis (e.g., Kurmayer et al., 2017) or cyanobacteria associated with the production of cyanotoxins, e.g. through isolated strains in the laboratory (Meriluoto et al., 2017, Appendix 2). To comprehensively gather information on all toxigenic cyanobacteria through HTS, this study used two primer systems. The first one targeted the amplification of the 16S rDNA V3–V4 region of bacteria in general, as described by Herlemann et al. (2011). The second primer system was specifically designed to amplify the 16S rDNA V3–V4 region of cyanobacteria, as established by Nübel et al. (1997) and Turner et al. (1999). Notably, both primer systems revealed comparable results, and a minor difference in cyanobacterial genera was observed.

The genus *Microcystis* was identified as the predominant MC producer among all MC-positive sites (A, B, C, G, H, I, J, K). Co-occurring toxigenic genera such as *Planktothrix* and *Dolichospermum* were typically found in lower abundances compared to *Microcystis*. However, their abundances may increase seasonally or under altered ecological conditions. A study conducted in 70 lakes in Finland (Rantala et al., 2006) found that eutrophication led to an increase in the frequency of the simultaneous occurrence of all three potentially MC-producing cyanobacteria, i.e., *Microcystis, Planktothrix*, and *Dolichospermum* (*Anabaena*). It is widely recognized that under different prevailing ecological conditions, other genera may exhibit dominance, i.e., *Dolichospermum* replacing *Microcystis* under nitrogen-limiting conditions. Therefore, solely relying on targeted genotype quantification through qPCR for *mcy*B in *Microcystis* and *Planktothrix* may be misleading and could underestimate the potential for MC production. In contrast, when monitoring the cyanobacterial community composition, a significantly broader range of information regarding potential MC producers was obtained. For example, through the HTS technique, monitoring encompassed not only planktonic genera but also benthic cyanobacteria growing in the littoral zone. In this example, most importantly, the toxigenic genera *Tychonema* and *Kamptonema* were also included in the analysis. In comparison with planktonic cyanobacteria, the formation of benthic cyanobacterial mats is less dependent on eutrophication. Such mats have also been observed even under oligotrophic conditions (Quiblier et al., 2013). It is likely that under oligotrophic conditions, favorable factors such as high water clarity and increased periods of high insolation (especially during spring and early summer in the northern hemisphere) contribute to the development of benthic mats which can grow up a thickness of several centimeters on the lake sediment (i.e., *Oscillatoria princeps* in Ossiachersee, Fresner et al., 1998). Later in the year, during the seasonal cycle, benthic cyanobacteria may form floating scums, often because of oxygen production during photosynthesis.

Numerous benthic cyanobacteria have been described to produce cyanotoxins, as evidenced by studies such as Wood et al. (2020). However, in many cases, the specific genes associated with toxin synthesis in these cyanobacteria have not yet been elucidated. For the genus *Kamptonema* (*Oscillatoria* PCC6506), the genes encoding anatoxin synthesis have been elucidated (Méjean et al., 2014). In addition, for *Tychonema bornetti* or *T. bourellyi*, gene fragments indicative of anatoxin synthesis have been sequenced (Shams et al., 2015). The occurrence of anatoxin production in benthic cyanobacterial mats has frequently been linked with incidents of poisoning in livestock and pets (Fastner et al., 2018; Wood et al., 2020). In this study, the four identified *Tychonema* ASVs (ASV866, ASV960, ASV1139 and ASV1409) each showed at least one substitution compared to the toxic *Tychonema* strains (NIVA-CYA 60, NIVA-CYA 96/3) published by Shams et al. (2015). Furthermore, the detected *Kamptonema* ASVs (ASV1390, ASV1443 and ASV3730) exhibited various substitutions when compared to anatoxin-producing strains such as *Kamptonema* PCC6506. Therefore, based on the finding of this study, the detected benthic taxa *Tychonema* and *Kamptonema* may not be classified as toxigenic. In conclusion, HTS enables the monitoring of potentially toxigenic cyanobacteria originating from the planktonic zone but also from benthic habitats.

### 4.2 Environmental parameters related to MC occurrence

Understanding the environmental factors that contribute to the occurrence of cyanobacterial harmful algal blooms (CHABs) is crucial, especially for the implementation of effective mitigation and management strategies. In general, standing freshwater waterbodies that exhibit elevated concentrations of total phosphorus (TP), above 20 μg L^−1^, high turbidity (Secchi depths <2 m), low water exchange rates, and thermal stratification are at greater risk of developing CHABs (Chorus and Welker, 2021). Extensive research has been conducted in the past to examine the influence of environmental parameters, such as water temperature and nutrient levels, on the production of different MC structural variants (Tonk et al., 2008; Van de Waal et al., 2010). However, due to the variability in geography, hydrology, and analytical methods used across various regions, it was difficult to establish general conclusions from the findings. For example, Su et al. (2015) identified cyanobacterial density, pH, TP, and water temperature as the key factors driving MC production. Their findings further demonstrate that MC-LR and MC-RR are the dominating MC structural variants during warmer months, whereas MC-YR is predominantly produced during colder months. In this study, the multivariate statistical analysis demonstrated positive relationships between MC occurrence with TChl and TP, whereas negative relationships were observed between MC occurrence and transparency (Fig. 5A). Despite the absence of any specific environmental parameter identified as a key factor in promoting the production of a specific MC structural variant, it was evident that the variability of MC structural variants was influenced by the characteristics of the study sites (Fig. 5B). Upon closer examination of the dominant cyanobacterial genotypes, it becomes apparent that sampling site J, which exhibited a predominance of variants MC-LR and MC-YR, was dominated by *Microcystis*-assigned ASV5. In contrast, sampling site I, which mostly produced variants MC-LR and MC-RR, was mainly dominated by *Microcystis*-assigned ASV19. This study did not investigate other environmental conditions such as water exchange rate, lake morphometry, thermal stratification, and the presence or absence of crustaceans and macrophytes, which could potentially influence cyanobacterial dominance (Dokulil and Teubner, 2000).

**Fig. 6:**
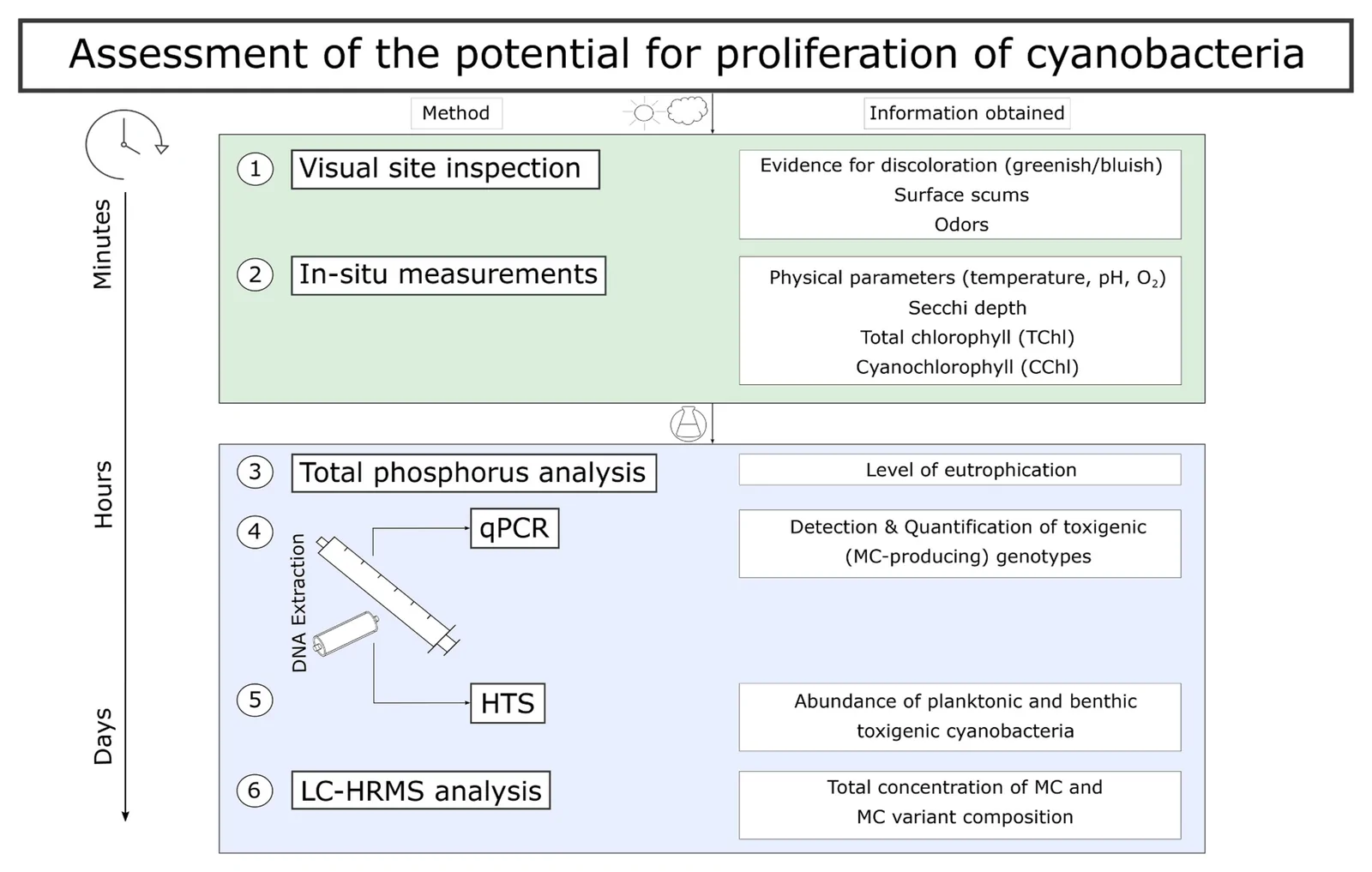
Proposed workflow to assess the potential for proliferation of cyanobacteria in bathing waters. A timeline indicates when information from the proposed methods becomes available. In the upper part of the illustration, in-situ measurements on-site are shown, whereas on the lower half, laboratory analyses are presented.

The findings of this study suggest that for an ideal risk assessment of CHABs in bathing water profiles, a comprehensive approach should be adopted. This approach should be based on (i) environmental parameters (e.g., trophic condition, water temperature), (ii) the use of qPCR to determine the MC-production potential of target species, and (iii) the analysis of the planktonic and benthic cyanobacterial community composition through HTS. By integrating these three proposed methods, a more accurate risk assessment of CHABs can be achieved (Fig. 6). Whilst the methods applied on-site allow an early warning for cyanobacterial mass proliferation, the confirmation of potential and actual cyanotoxin occurrence is obtained through the proposed laboratory techniques. Regarding Table S1, five sampling sites (A, G, H, I, J) have been defined as highly toxic risk sites by their corresponding bathing water profiles. Nevertheless, MC occurred in 19 samples which originated from eight sampling sites (A, B, C, G, H, I, J, K), i.e., the toxic sampling sites C and K were evaluated with “moderate risk” and showed MC once (at the end of July), with 0.1 and 0.11 μg L^−1^, respectively. Additionally, sampling site B was classified as a “no risk” site, although MC < LOQ was detected.

Aside from the parameters TP, TChl, and Secchi depth, used for bathing water site risk assessment, a significant relationship between MC-positive samples with increased water temperature (Fig. 5A) was found in this study. Throughout the study period, in the summer of 2020, there were large fluctuations in weather conditions, including the absence of prolonged heat waves and an average increase of 25 % in precipitation (ZAMG, 2020). In temperate climatic conditions, the proliferation of cyanobacteria is often linked with higher water temperatures (above 20 °C) (Jöhnk et al., 2008; Mantzouki et al., 2018; O’Neil et al., 2012; Walls et al., 2018). In the context of a warming climate,Central Europe can expect a rise in the occurrence of prolonged heat waves and decreased precipitation, as experienced during the summer of 2022. Thus, it is plausible to anticipate a more frequent occurrence of CHABs at bathing sites during periods of more stable summer conditions (Visser et al., 2016). Should this scenario become true, there would be a need for the real-time monitoring of water temperature to effectively recognize and respond to CHAB occurrence triggered by actual temperature increases.

### 4.3 Methodological considerations in monitoring

With regard to health risk management, a list of guideline concentrations has been proposed, which remains relevant regardless of whether water authorities are empowered to take action or not (i.e., Chapter 5 in Chorus and Welker, 2021). However, for several cyanotoxins, such as MCs over 200 structural variants are known, which neither have been characterized in toxicological terms nor can be all analyzed using the available analytical standards. Therefore, to establish the most current guidelines, the overall MC toxicity has been assessed by calculating their equivalents based on MC-LR. In addition, due to the lack of oral toxicity data for other MC-congeners, it is recommended to apply the guideline values to the total MCs present (Chorus and Welker, 2021). Considering the impracticability of analyzing all potential structural variants, alert levels for cyanobacterial biomass have been suggested as a proxy for the possible occurrence of cyanotoxin concentrations reaching the guideline values (Chorus and Bartram, 1999; Chorus and Welker, 2021; World Health Organization, 2021). During this study, the observed *Microcystis* cell densities (in cell equivalents of DNA) only rarely exceeded the frequently cited alert level 2 of 100,000 cells mL^−1^, implying that assessing health risks and regular monitoring are recommended (Fig. 6.3 in Chorus and Bartram, 1999). Accordingly, 8 mm^3^ L^−1^ of cyanobacterial biomass or 24 μg Chl-a L^−1^ might translate into 24 μg L^−1^ of total MC (Table 5.2, Chapter 5.2 in Chorus and Welker, 2021). In addition to the information on MC concentration, quantitative information on cell numbers as inferred by qPCR or HTS will provide relevant complementary information to decide on potential protective actions.

Currently, in a few EU member states, HTS and/or qPCR are used for the surveillance of national bathing waters regarding cyanobacterial risk assessment and monitoring, such as in, Belgium (Van Hassel et al., 2022), Bulgaria (Radkova et al., 2020), Poland (Głowacka et al., 2011), and Slovenia (Zupančič et al., 2021). However, most publications use cyanobacterial monitoring programs based on cell counting, microscopic analyses, and/or direct MC measurements, i.e., Germany (Frank, 2002), Greece (Christophoridis et al., 2018; Gkelis and Zaoutsos, 2014), Italy (Funari et al., 2017) and the Netherlands (Schets et al., 2020). This study shows that based on HTS and qPCR quantification of gene loci indicative of MC production, the MC production potential for bathing waters can be evaluated and if necessary, even corrected. Thus, in combination with the monitoring of standard environmental parameters, the use of molecular high-throughput tools is considered a promising and time-efficient alternative to the established microscopic enumeration protocols.

## 5 Conclusions and outlook

In this study, we propose an improved workflow for evaluating the risk of harmful cyanobacterial algal blooms in EU bathing water sites. The proposed workflow involves integrating environmental monitoring data (such as water temperature and Secchi depth) and essential parameters (such as total chlorophyll (TChl) and total phosphorus (TP) with high-throughput sequencing (HTS) and quantitative polymerase chain reaction (qPCR) techniques. Among the dominant microcystin (MC) producers observed in this study, *Microcystis* and *Planktothrix* were most prevalent, although the latter was only present in limited numbers of samples. Within the genus *Microcystis*, the *mcy*B genotypes, which serve as indicators of MC production, formed a stable subpopulation that increased linearly in correlation with the total *Microcystis* population. Notably, the HTS read counts assigned to *Microcystis* and *Planktothrix* correlated with the corresponding qPCR estimates of genotype abundance, confirming the suitability of using (semi-) quantitative sequencing through HTS. The application of in-situ measurements allows an early warning for a cyanobacterial bloom, whereas the proposed laboratory analysis allows the confirmation or revision of the actual cyanotoxin occurrence. In addition to the observed increases in TP and, TChl reduced transparency, a rise in water temperature, as well as cyanobacterial HTS read numbers and *Microcystis* cell numbers, were associated with positive MC samples. In combination with the monitoring of standard environmental parameters, the integration of HTS and qPCR techniques is regarded as highly beneficial for the timely identification of health risks to recreational users, as mandated by the Bathing Water Directive (BWD).

## Supplementary Material

Supplementary data to this article can be found online at https://doi.org/10.1016/j.scitotenv.2023.165934.

Supplementary

## Figures and Tables

**Table 1:** List of primers and probes used for HTS and qPCR in this study.

Target gene	Primer name	Sequence 5’to 3’	PCR product size	Use	Source
V3-V4 bacterial 16S rRNA	Bakt_341F Bakt 805R	CCTACGGGNGGCWGCAG GACTACHVGGGTATCTAATCC	500 bp	Illumina amplicon PCR	Herlemann et al. (2011)
V3-V4 cyanobacterial 16S rRNA	515F CYA781R(a) CYA781R(b)	GTGCCAGCMGCCGCGGTAA GACTACTGGGGTATCTAATCCCATT GACTACAGGGGTATCTAATCCCTTT	362 bp	Illumina amplicon PCR	Turner et al. (1999) Nübel et al. (1997)
PC-IGS *(Microcystis)*	188F 254R TaqMan probe PC Mc	GCTACTTCGACCGCGCC TCCTACGGTTTAATTGAGACTAGCC CCGCTGCTGTCGCCTAGTCCCTG	66 bp	qPCR	Kurmayer and Kutzenberger (2003)
*mcyB (Microcystis)*	30F 108R TaqMan probe *mcy*B Mc	CCTACCGAGCGCTTGGG GAAAATCCCCTAAAGATTCCTGAGT CACCAAAGAAACACCCGAATCTGAGAGG	78 bp	qPCR	Kurmayer and Kutzenberger (2003)
PC-IGS *(Planktothrix)*	PC-IGS_F PC-IGS_R TaqMan probe PC Pl	GAGCAGCACTGAAATCCAAG GCTTTGGCTGCTTCTAAACC TTTGGCTTGACGGAAACGACCAA	72 bp	qPCR	Schober and Kurmayer (2006)
*mcyB (Planktothrix)*	Pl *mcy*B_F Pl *mcy*B_R TaqMan probe *mcy*B Pl	ATTGCCGTTATCTCAAGCGAG TGCTGAAAAAACTGCTGCATTAA TTTTTGTGGAGGTGAAGCTCTTTCCTCTGA	76 bp	qPCR	Ostermaier and Kurmayer (2010)

**Table 2:** List of cyanobacterial taxa recorded most frequently by HTS from 16 EU-bathing waters located in the east of Austria during the bathing season in the summer of 2020. Two different primer pairs (bacteria general or cyanobacteria specific) have been used. Only the taxa with total read numbers ≥0.1 % have been included. Potential MC-producers are printed in bold.

Cyanobacterial genus (SILVA DB)	Sum of reads (bacterial)	Number of assigned ASVs (bacterial)	Sum of reads (cyanobacterial)	Number of assigned ASVs (cyanobacterial)
*Cyanobium*	104,169 (56 %)	121	89,145 (51 %)	112
*Microcystis*	**19,304 (10 %)**	**7**	**26,704 (15 %)**	**9**
*Snowella*	12,757 (7 %)	14	13,200 (8 %)	15
*Pseudanabaena*	11,110 (6 %)	21	6082 (4 %)	20
*Prochlorothrix*	9469 (5 %)	4	8721 (5 %)	2
*Planktothrix*	**9351 (5 %)**	**6**	**6568 (4 %)**	**4**
*Dolichospermum*	**9178 (5 %)**	**9**	**5968 (3 %)**	**10**
*Aphanizomenon NIES81*	**5246 (3 %)**	**17**	**5816 (3 %)**	**15**
*Nodosilinea*	1963 (1 %)	6	1077 (1 %)	6
*Tychonema*	1284 (1 %)	4	1840 (1 %)	4
*Aphanizomenon MDT14a*	**1252 (1 %)**	**3**	**6340 (4 %)**	**3**
*Synechococcus PCC-7502*	219 (0.1 %)	1	219 (0.1 %)	1
*Cuspidothrix LMECYA 163*	144 (0.1 %)	3	188 (0.1 %)	2
*Kamptonema. PCC-6407*	138 (0.1 %)	3	155 (0.1 %)	4
*Leptolyngbya SAG 2411*	135 (0.1 %)	1	158 (0.1 %)	1
*Planktothricoides SR001*	108 (0.1 %)	1	112 (0.1 %)	1

## Data Availability

Data will be made available on request.

## Highlights

- Microcystin (MC)-producing potential and MC was evaluated for EU-bathing waters.
- Combining high-throughput sequencing (HTS) and qPCR predicted MC most reliably.
- *Microcystis* and *Planktothrix* assigned HTS read numbers correlated with qPCR.
- Improved risk assessment via environmental parameters, HTS and qPCR.
