# Supplementary material for: Quantitative relationships among high-throughput sequencing, cyanobacteria toxigenic genotype abundance and microcystin occurrence in bathing waters
Source: Sci Total Environ. Author manuscript; Available in PMC 2026 Oct 6. (PMC7619507; doi:10.1016/j.scitotenv.2023.165934)
Supplement: Supplementary file 1 [file EMS217545-supplement-Supplementary.docx]

Supplementary Information

Quantitative relationships between high-throughput sequencing, cyanobacteria toxigenic genotype abundance and microcystin occurrence in bathing waters

Magdalena Purker ^1,2,6^, Stefanie Dobrovolny ^3^, Michaela Kreuml ^4^, Peter Hufnagl ^1^, Alexander Indra ^5^ and Rainer Kurmayer ^6^

^1^ Austrian Agency for Health and Food Safety, Institute for Medical Microbiology and Hygiene - Center for Anthropogenic Infections, Department of Clinical Molecular Biology, Währinger Straße 25a, 1090 Vienna, Austria

^2^ Austrian Agency for Health and Food Safety, Institute for Medical Microbiology and Hygiene - Center for Anthropogenic Infections, Department of Water and Hygiene, Währinger Straße 25a, 1090 Vienna, Austria

^3^ Austrian Agency for Health and Food Safety, Institute for Food Safety, Department of Molecular Biology and Microbiology, Spargelfeldstraße 191, 1220 Vienna, Austria

^4^ Austrian Agency for Health and Food Safety, Institute for Hydroanalytics, Wieningerstraße 8, 4020 Linz, Austria

^5^ Austrian Agency for Health and Food Safety, Institute for Medical Microbiology and Hygiene, Währinger Straße 25a, 1090 Vienna, Austria

^6^ Universität Innsbruck, Research Department for Limnology, Mondseestrasse 9, 5310 Mondsee, Austria and Universität Innsbruck, Innrain 52, 6020 Innsbruck

HTS reactions

For the bacterial PCR assay the reaction mix was consisting of 12.5 µL KAPA HiFi HotStart ReadyMix (Roche, Basel, Switzerland), 0.05 µL forward Primer (c = 100 µM), 0.05 µL reverse Primer (c = 100 µM), 7.4 µL ddH_2_O and 5 µL of DNA. The bacterial amplicon PCR was performed under the following conditions: 3 min, 95°C; 30 s, 95°C; 30 s, 55°C; 30 s, 72°C; 30 cycles; 5 min, 72°C. The reaction mix for the cyanobacterial PCR assay consisted of 12.5 µL HotStartTaq Master Mix (Qiagen, Hilden, Germany), 0.1 µL forward Primer (c = 100 µM), 0.05 µL reverse Primer a (c = 100 µM), 0.05 µL reverse Primer b (c = 100 µM), 1.5 µL MgCl_2_ (c = 25 mM), 5.8 µL ddH_2_O and 5 µL of DNA. For the cyanobacterial amplicon the PCR conditions were the following: 15 min, 95°C; 30 s, 95°C; 30 s, 65°C; 30 s, 72°C; 30 cycles; 10 min, 72°C.

qPCR calibration curves

Cyanobacteria strains were grown semi-continuously under maximum growth rate conditions in BG11 medium at 20°C with 50 µE m^-2^ s^-1^ irradiation (12:12h light and dark cycle). During cell harvest for DNA extraction aliquots (1.8 mL) were fixed with formaldehyde for microscopic cell counting. One mL of the fixed cells were filtered on a 0.22 µm membrane filter, a drop of immersion oil was added and, finally, covered by a cover slip. For counting, a fluorescence-microscope (Olympus BX 53 equipped with an Olympus U-RFL-T mercury burner USH-1030L as light source) in combination with a Cy5 filter for auto fluorescence was used. Thirty random pictures were made with a Nikon DS-Fi3 camera throughout the filter area. Coccal cells (e.g. *Microcystis aeruginosa* strain Hofbauer) were counted manually, filamentous cells (e.g. *Planktothrix* strain NIVA-CYA 126/8) were counted using the software PlanktothriQuant (Zeder et al., 2010). Biovolume of CYA 126/8 was calculated by following the formula: V = r^2^*×*π*×*h, whereby r^2^ corresponded to the measured mean width and h was represented by the measured length of the *Planktothrix* filaments (Teubner et al. 2022, Rantala et al. 2017).

Comparing diversity estimates from HTS using either bacterial or cyanobacterial primers

In general, the cyanobacterial community richness estimates calculated from sequencing results using either bacterial or cyanobacterial primers varied (Figure S4). At the start of the sampling season (early June until early July – Wilcoxon Signed-Rank Test; p < 0.01), i.e. more than twice the amount of cyanobacterial ASVs were detected using the cyanobacterial primers. Interestingly, from the fourth sampling date onwards (end of July), no significant difference could be detected anymore (Wilcoxon Signed-Rank Test; p > 0.05). Thus, while bacterial primers underestimated cyanobacteria diversity as long as abundance of total cyanobacteria remained low (early June – Mid July), bacterial primers and cyanobacterial primers revealed more comparable results when cyanobacteria became dominant during the second half of the bathing season (see Figure S5).

Bacterial community composition

Overall, the most prominent bacterial phyla within the observed waterbodies included Actinobacteriota (224742 reads), Proteobacteria (213189 reads) and Cyanobacteria (149604 reads), corresponding to 30%, 28% and 20% of all bacterial reads, respectively. Throughout the bathing season, an increase in cyanobacteria proportion at the expense of other heterotrophic bacterial phyla was observed (Fig. S5). In early-June, the dominating phyla were Actinobacteriota (53885 reads, 36%), followed by Proteobacteria (38926 reads, 26%) and Campylobacterota (21973, 15%). Only 11% of the reads (15852 reads) were assigned to cyanobacteria. This phylum composition remained until end of June, i.e. approx. 30% of the reads were assigned to Proteobacteria (47162 reads) and Actinobacteriota (45492 reads) while Campylobacterota occurred with 28909 reads (19%). As little as 12456 reads were assigned to Cyanobacteria (8%). In contrast, at the third sampling date in early-July the abundance of Cyanobacteria started to increase (25961 reads, 17%) as well as Proteobacteria sequences increased to 53529 reads (36%), while Actinobacteriota (37288, 25%) and Campylobacterota (12395, 8%) rather decreased. At the fourth sampling date at the end of July, Cyanobacteria constituted the second most abundant phylum with 40200 reads (27%), second to Actinobacteriota (55108, 37%). Finally, during mid of August, Cyanobacteria were found most abundant resulting in 55135 reads (37%), followed by Proteobacteria (41013, 27%) and Actinobacteriota (32860, 22%).

Table S1: Description of the sampled EU-bathing waters, based on their bathing water profiles (BWD, 2006/7/EC). Letters A - P indicate the respective sites.

| **Bathing site** | **Description of lake** | **Level of eutrophication** | **CHAB classification based on** | **Risk of CHAB occurrence** |
| --- | --- | --- | --- | --- |
| A | heavily modified | hypertrophic | Nutrients, Chl-a | high risk |
| B | semi natural | unknown | Visual impression | no risk |
| C | semi natural | eutrophic | Nutrients, Chl-a and microscopy | moderate risk |
| D | semi natural | eutrophic | Nutrients, Chl-a and microscopy | moderate risk |
| E | semi natural | unknown | Visual impression | no risk |
| F | semi natural | mesotrophic | Nutrients, visual impression | no risk |
| G | semi natural | hypertrophic | Nutrients, Chl-a and microscopy | high risk |
| H | semi natural | unknown | Visual impression | high risk |
| I | reservoir | eutrophic - hypereutrophic | Nutrients, cyanotoxins | high risk |
| J | semi natural | eutrophic | Nutrients, cyanotoxins | high risk |
| K | artificial | eutrophic | Nutrients, Chl-a and microscopy | moderate risk |
| L | semi natural | mesotrophic | Nutrients, Chl-a and microscopy | moderate risk |
| M | reservoir | eutrophic | Outdated limnological study | moderate risk |
| N | semi natural | eutrophic | Outdated limnological study | moderate risk |
| O | semi natural | oligo- mesotrophic | Visual impression | moderate risk |
| P | semi natural | eutrophic | Nutrients, Chl-a and microscopy | moderate risk |

Table S2: List of the four analyzed MC structural variants in bathing waters: Retention time for HPLC, detection limits (LOD) and quantification limits (LOQ) for the analyzed MC variants.

| **Microcystin variant** | **Retention time [min]** | **LOD [µg L^-1^]** | **LOQ [µg L^-1^]** |
| --- | --- | --- | --- |
| MC-LR | 10.54 | 0.022 | 0.080 |
| MC-RR | 10.12 | 0.024 | 0.088 |
| MC-YR | 10.36 | 0.023 | 0.084 |
| [D-Asp^3^,(E)-Dhb^7^]-MC RR | 10.12 | 0.023 | 0.086 |

Table S3: Statistical overview from standard curves for the qPCR assays. AE = amplification efficiency in %.

| **Culture** | **Linear regression** | **r^2^** | **AE** |
| --- | --- | --- | --- |
| ***M. aeruginosa*** Hofbauer (PC) | y = 37.5515 – 3.5979 log10(x) | 1 | 90.19 |
| ***M. aeruginosa*** Hofbauer (*mcy*B) | y = 44.5483 – 3.5711 log10(x) | 0.959 | 90.56 |
| CYA126/8 (PC) | y = 20.1703 – 3.7860 log10(x) | 0.998 | 83.71 |
| CYA126/8 (*mcy*B) | y = 19.8561 – 4.0316 | 0.997 | 77.03 |

Table S4: Ct-values representing LODs and LOQs for the qPCR assays.

| **Culture** | **LOD** | **LOQ** |
| --- | --- | --- |
| ***M. aeruginosa*** Hofbauer (PC) | 35.81 | 35.81 |
| ***M. aeruginosa*** Hofbauer (*mcy*B) | 37.36 | 37.36 |
| ***P. agardhii*** CYA126/8 (PC) | 38.03 | 38.03 |
| ***P. agardhii*** CYA126/8 (*mcy*B) | 34.40 | 34.40 |

Figure S1: Standard curves for each qPCR assay and culture.

Figure S2: Rarefaction curves of samples amplified with bacterial primers during (A) Early-June, (B) Mid-June, (C) Early-July, (D) End-July, (E) Mid-August. The rarefied threshold was set to 9484 sequences (indicated by the black dotted line).

Figure S3: Rarefaction curves of samples amplified with cyanobacterial primers during (A) Early-June, (B) Mid-June, (C) Early-July, (D) End-July, (E) Mid-August. The rarefied threshold was set to 2519 sequences (indicated by the black dotted line).

Figure S4: Chao1 richness index calculated from HTS using either bacterial or cyanobacterial primers during five different sampling dates. For comparison, the same rarefying threshold for both datasets (2519 reads) was chosen.

Figure S5: Bacterial community distribution at phylum level recorded from 16 EU-bathing waters located in the east of Austria during the bathing season in the summer of 2020. For clarity rare phyla with a frequency below 50 reads have been collected under “other”.

Figure S6: Relationship between PC and *mcy*B genotypes (in biovolume) for *Planktothrix*. N = 80; Spearman`s rho = 0.59, p < 0.001.

Figure S7: Relationship between MC concentration and *mcy*B genotype of *Microcystis*. N = 64; Spearman`s rho = 0.68, p < 0.001.

Figure S8: Linear correlation between TChl and TP, P < 0.001 (A). Linear correlation between CChl and TP, p < 0.001 (B). Linear correlation between TChl and sum of cyanobacterial read numbers (bacterial Primers), p < 0.001 (C). Linear correlation between CChl and sum of cyanobacterial read numbers (bacterial Primers), p < 0.001 (D).

Figure S9: Linear correlation between MC concentration and CChl. N = 64; Spearman`s rho: 0.59, p < 0.001.

References

Rantala-Ylinen, A., H. Savela, K. Sivonen & R. Kurmayer, 2017. Quantitative PCR. In Kurmayer, R., K. Sivonen, A. Wilmotte & N. Salmaso (eds) Molecular tools for the detection and quantification of toxigenic cyanobacteria. John Wiley & Sons Ltd., Cichester, West Sussex, UK, 205-239.

Teubner, K., Dokulil, M. T., & Kurmayer, R. (2022). Eutrophierung, toxische Cyanobakterien am Beispiel des urbanen Donau-Altarmgewässers Alte Donau und des alpinen Mondsees. In A. H. Farnleiter, A. K. T. Kirschner, C. Frick, P. Proksch, W. Vogl, U. Schauer, B. Schrammel, & F. Zibuschka (Eds.), ÖWAV-Arbeitsbehelf 52, Teil 2: Fallstudien zur Illustration der neuen diagnostischen-analytischen Möglichkeiten (pp. 35–45).

Zeder, M., Van Den Wyngaert, S., Köster, O., Felder, K. M., & Pernthaler, J. (2010). Automated quantification and sizing of unbranched filamentous cyanobacteria by model-based object-oriented image analysis. *Applied and Environmental Microbiology*, *76*(5), 1615–1622. https://doi.org/10.1128/AEM.02232-09
